# miR-376a-3p and miR-376b-3p overexpression in Hutchinson-Gilford progeria fibroblasts inhibits cell proliferation and induces premature senescence

**DOI:** 10.1016/j.isci.2022.103757

**Published:** 2022-01-10

**Authors:** Diane Frankel, Valérie Delecourt, Elva-María Novoa-del-Toro, Jérôme D. Robin, Coraline Airault, Catherine Bartoli, Aurélie Carabalona, Sophie Perrin, Kilian Mazaleyrat, Annachiara De Sandre-Giovannoli, Frederique Magdinier, Anaïs Baudot, Nicolas Lévy, Elise Kaspi, Patrice Roll

**Affiliations:** 1 Aix Marseille Univ, APHM, INSERM, MMG, Hôpital la Timone, Service de Biologie Cellulaire, 27 Bd Jean Moulin, Marseille, France; 2 Aix Marseille Univ, INSERM, MMG, Marseille, France; 3 ProGeLife, Marseille, France; 4 Aix Marseille Univ, APHM, INSERM, MMG, Hôpital la Timone, Département de Génétique Médicale, Biological Resource Center (CRB-TAC), Marseille, France

**Keywords:** Biological sciences, Molecular biology, Cell biology

## Abstract

Hutchinson-Gilford progeria syndrome (HGPS) is a rare genetic disorder, in which an abnormal and toxic protein called progerin, accumulates in cell nuclei, leading to major cellular defects. Among them, chromatin remodeling drives gene expression changes, including miRNA dysregulation. In our study, we evaluated miRNA expression profiles in HGPS and control fibroblasts. We identified an enrichment of overexpressed miRNAs belonging to the 14q32.2-14q32.3 miRNA cluster. Using 3D FISH, we demonstrated that overexpression of these miRNAs is associated with chromatin remodeling at this specific locus in HGPS fibroblasts. We then focused on miR-376b-3p and miR-376a-3p, both overexpressed in HGPS fibroblasts. We demonstrated that their induced overexpression in control fibroblasts decreases cell proliferation and increases senescence, whereas their inhibition in HGPS fibroblasts rescues proliferation defects and senescence and decreases progerin accumulation. By targeting these major processes linked to premature aging, these two miRNAs may play a pivotal role in the pathophysiology of HGPS.

## Highlights

• Several miRNAs are deregulated in HGPS fibroblasts compared with controls
• Progerin leads to overexpression of miRNAs belonging to the 14q32.2-14q32.3 cluster
• miR-376a and miR-376b overexpression decreases cell proliferation and increases senescence

## Graphical abstract

**Figure:**
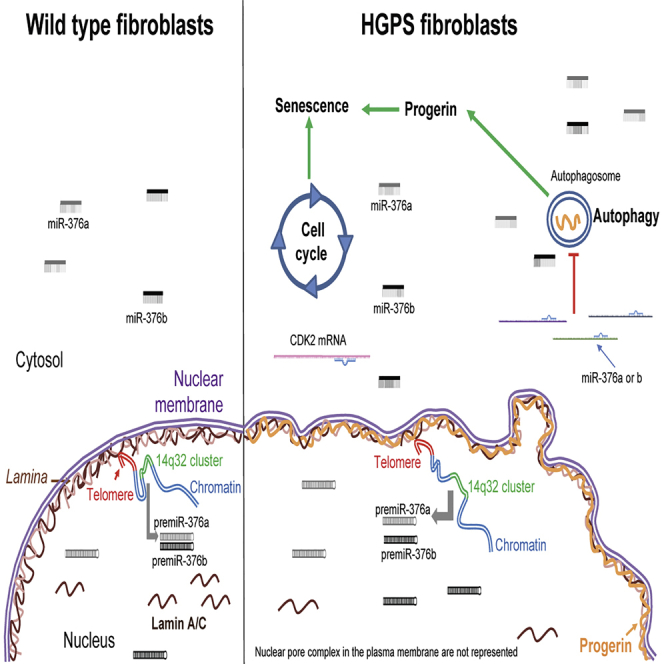

## Introduction

Hutchinson-Gilford progeria syndrome (HGPS; OMIM #176670) is a rare genetic disease affecting approximately one in 8–10 million children. Children with HGPS appear healthy at birth and progressively develop clinical features of premature and accelerated aging within the first years of life. Myocardial infarction due to systemic arterial alterations and stiffness is the most frequent cause of death, which occurs at a mean age of 14.6 years (Gordon et al., 2014). Classic HGPS is primarily caused by a *de novo* mutation (c.1824C > T, p.G608G) in exon 11 of the *LMNA* gene, encoding nuclear A-type lamins (De Sandre-Giovannoli et al., 2003; Eriksson et al., 2003). This mutation leads to the production of a 50 amino acid internally truncated, farnesylated prelamin A called progerin. This toxic protein affects the structure and functions of the nucleus and triggers multiple deleterious effects in HGPS cells. For instance, progerin induces mechanical defects, slows proliferation and the cell cycle, alters chromatin organization, and delays DNA repair, enhancing senescence (Cau et al., 2014). Moreover, progerin accumulates in cells, likely due to defective clearance, which might be linked with autophagy (Cenni et al., 2011; DuBose et al., 2018).

Identification in 2012 of the major role of the miR-9 microRNA (miRNA) in HGPS led to a better understanding of the disease (Jung et al., 2012; Nissan et al., 2012). MiRNAs are small noncoding RNAs (19–25 nucleotides) that control gene expression by pairing in most cases, to the 3′UTR of messenger RNAs (mRNAs), leading to translational repression or mRNA degradation (Kim and Nam, 2006). MiR-9, which is highly expressed in neurons, binds to the 3′UTR of wild-type prelamin A and progerin transcripts, triggering their degradation, thus participates to the protection of neural cells from progerin accumulation in affected children (Jung et al., 2012; Nissan et al., 2012). Several other miRNAs have been identified as dysregulated in laminopathies and are associated with deleterious effects (Frankel et al., 2018). Using *Zmpste24*^−/−^ progeria mouse model, only four miRNAs have been described (Mariño et al., 2010; Ugalde et al., 2011; Xiong et al., 2015; Zhang et al., 2017). To our knowledge, no global miRNA profiling has been reported in human HGPS fibroblasts.

To bridge this gap, we performed global miRNA profiling of 375 miRNAs in five HGPS and five control (wild-type) fibroblasts. We identified eight overexpressed miRNAs, among which seven belonged to the 14q32.2–14q32.3 miRNA gene cluster. At this locus, expression changes correlate with chromatin modifications. By focusing on miR-376b-3p and miR-376a-3p, two miRNAs from this cluster belonging to the same family, we discovered that they may play a role in cell proliferation and progerin clearance defects, ultimately leading to the premature senescence, which participates to HGPS pathophysiology.

## Results

### Profiling miRNA expression in HGPS fibroblasts

We profiled the expression of 375 miRNAs using a quantitative RT-PCR (RT-qPCR) approach in five HGPS compared with five wild-type (controls) fibroblasts at passage 12 ± 2 (P12 ± 2). To evaluate miRNA expression evolution in the context of “cellular aging” (Benson et al., 2010), we also included a later passage (P20 ± 1) for two controls (C1 and C5) and three HGPS fibroblasts (HGPS2, HGPS3 and HGPS4) (Table S1). Of note, for HGPS1 and HGPS5, fibroblasts stopped proliferating before P20 due to premature senescence. Based on recorded quantification cycle (Cq) values, 188 miRNAs out of the 375 tested were considered expressed (see STAR Methods section) in our samples and were retained for further analysis (https://doi.org/10.6084/m9.figshare.9642803.v1).

**Figure 1:**
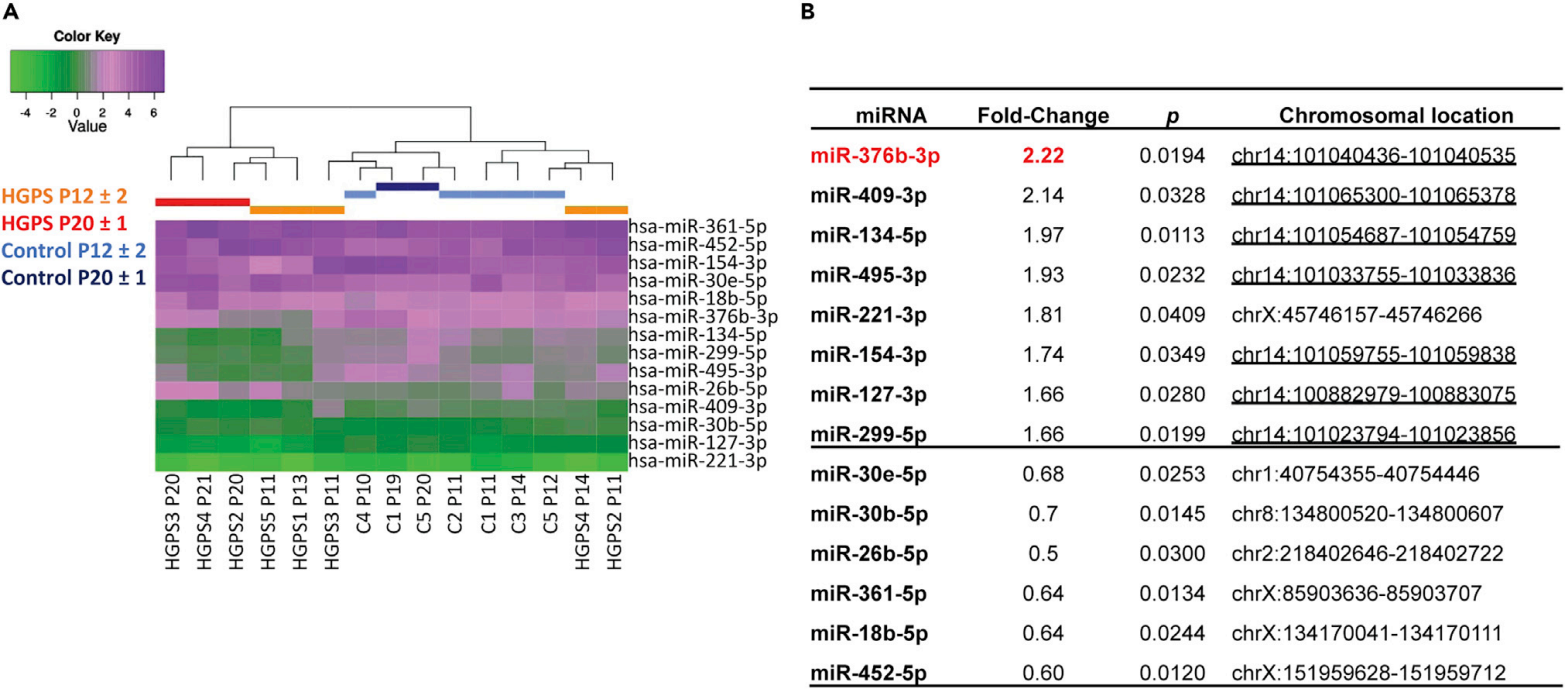
miRNA expression profile in HGPS fibroblasts (A) Unsupervised hierarchical clustering of the 14 differentially expressed miRNAs in HGPS and control fibroblasts and without deregulation in controls. Each column represents the miRNAs expression profile in HGPS and control samples (ΔCq, see https://doi.org/10.6084/m9.figshare.9642803.v1.). Color scale corresponds to the miRNA expression level represented by ΔCq values. (B) List of the 14 dysregulated miRNAs between HGPS and control fibroblasts. The fold-change (mean fold-change of HGPS samples), the p-value (**t-test**), and the chromosomal location of each miRNA are indicated.

Among the 188 miRNAs expressed, statistical analyses led to the identification of 14 miRNAs that were differentially expressed between HGPS and control cells (p< 0.05, t-test, see STAR Methods section). Unsupervised hierarchical clustering of these 14 differentially expressed miRNAs separated samples into two different groups (Figure 1A). The first group included three HGPS fibroblasts at late passage P20 ± 1 and two HGPS fibroblasts at early passage P12 ± 2, whose proliferation ceased before P20. The second group included all controls and the three other HGPS samples at P12 ± 2. This clustering suggests that, depending on the patient, miRNA profiling of HGPS fibroblasts at early passages (P12 ± 2) could be similar either to controls or alternatively to other HGPS fibroblasts at later passages (P20 ± 1). Among the 14 differentially expressed miRNAs, almost all upregulated miRNAs (7 out of 8) and none of the downregulated miRNAs (0 out of 6) belonged to a large gene cluster located in the 14q32.2-14q32.3 region (Figure 1B, p< 0.05, hypergeometric test), suggesting a global dysregulation of this cluster.

### miRNA dysregulation in the 14q32 region is associated with epigenetic changes

**Figure 2:**
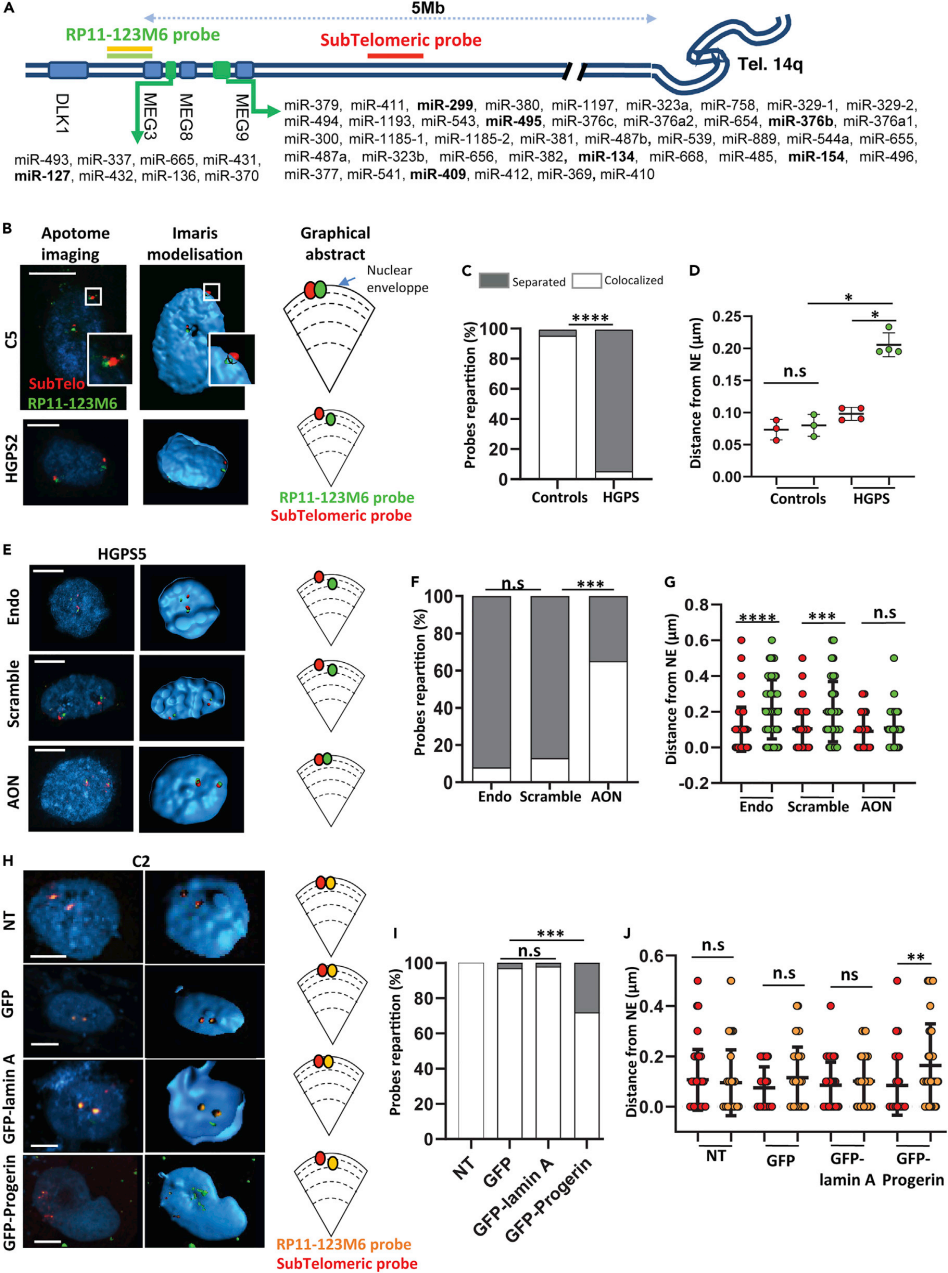
Epigenetic modifications in HGPS fibroblasts (A) Schematic map of a part of 14q chromosome containing the cluster of miRNA genes. The RP11-123M6 probe (green) stains a part of the cluster of miRNA genes; a subtelomeric probe (red) targets the most unique telomeric region of chromosome 14q. MiRNAs in bold are differentially expressed in our miRNome study. (B, E, and H) Representative images of 3D-FISH obtained with an apotome microscope (Carl Zeiss Microimaging, Jena, Germany) and processed with IMARIS for n ≈ 30 nuclei per sample. Schematic distribution of the probes relative to the nuclear envelope is represented. (B) C5 and HGPS2 fibroblasts; (E) HGPS5 fibroblasts transfected with endoporter only (endo), scramble AON (scramble), or exon 10 and exon 11 AON (AON); (H) C2 transfected with a plasmid expressing GFP-progerin or GFP-wild-type lamin A (GFP-lamin A) or only GFP (GFP) or not transfected (NT). Representative images are shown; scale bar represents 5 μm (C, F, and I) Percentage of separated and colocalized probes in (C) control fibroblasts (n = 3) or HGPS fibroblasts (n = 4) (Chi-square test); (F) HGPS5 fibroblasts transfected with endoporter only (Endo), scramble AON (Scramble), or exon 10 and exon 11 AON (AON) (Chi-square test); (I) C2 transfected with a plasmid expressing GFP-progerin or GFP-wild-type lamin A (GFP-lamin A), or only GFP (GFP) or not transfected (NT) (Chi-square test). (D, G, and J) Distance between the probes and the nuclear envelope (NE) in (D) control fibroblasts (mean of 30 nuclei from three control fibroblasts) or HGPS fibroblasts (mean of 30 nuclei from four HGPS fibroblasts) (Mann Whitney test, see Figure S2B for details). (G) In HGPS5 fibroblasts transfected with endoporter (Endo) or with scramble AON (Scramble) or with exon 10 and 11 AON (AON) (ANOVA test). (J) C2 transfected with plasmid expressing GFP-progerin or GFP-wild-type lamin A (GFP-lamin A) or only GFP (GFP) or not transfected (NT) (ANOVA test). Data are represented as mean ± SEM (∗p < 0.05, ∗∗p < 0.01, ∗∗∗p < 0.001, and ∗∗∗∗p < 0.0001)

A-type lamins are involved in chromatin organization, and contact-repressed genomic regions are called lamin-associated domains (LADs) (Guelen et al., 2008; Robin and Magdinier, 2016). Because the 14q32.2-14q32.3 miRNA gene cluster (hereafter called the 14q32 cluster) is localized in the vicinity of one of these LADs (Rao et al., 2014) (Figure S1A), we wondered whether this dysregulation might result from local chromatin remodeling. Therefore, we investigated the topology of the locus by three-dimensional fluorescence *in situ* hybridization (3D-FISH) using a DNA probe spanning the region of interest (RP11-123M6) and a probe targeting the most unique telomeric region of chromosome 14q (subtelomeric probe, Figure 2A). The 14q32 cluster is localized 5 Mb from the telomeres. The subtelomeric probe (<1 Mb from telomeres) is used to evaluate changes in the position of the 14q32 cluster relative to the telomeric region and to its position to the nuclear periphery (Wood et al., 2014).

We performed 3D-FISH in four HGPS fibroblasts (HGPS2, HGPS4, HGPS5, and HGPS6) and in three control fibroblasts (C1, C2, and C5) (Figure 2B and Figure S1B). In control fibroblasts, we showed that the 14q32 cluster is localized close to the nuclear envelope. The probe spanning the 14q32 cluster and the subtelomeric probe were colocalized in 95.6% of nuclei (Figure 2C). In HGPS fibroblasts, the distance between the two probes was increased, with only 5.75% of nuclei presenting colocalized probes (Figures 2B and 2C, p< 0.0001, Fisher’s test; Figure S1C, Mann Whitney test), as was the distance between the nuclear rim and the 14q32 probe (Figure 2D, p< 0.05, Mann-Whitney test).

To determine whether the presence of progerin is the cause of this cluster delocalization, we first transfected HGPS5 fibroblasts with antisense morpholinos (AON) targeting exons 10 and 11 of the *LMNA* gene, known to reduce progerin production (Harhouri et al., 2016). As expected, only 32% of HGPS5 nuclei expressed progerin after transduction of AON, as opposed to 86% and 90% under Endoporter and scramble conditions, respectively (Figure S1D). In HGPS cells, AON transfection restored colocalization between the two probes and their proximity to the nuclear envelope compared with control conditions (scramble and Endoporter, Figures 2E–2G), highlighting the direct link between progerin production and changes in the topology of this locus.

We challenged these results by reciprocally overexpressing progerin in control fibroblasts to evaluate the impact on chromatin conformation. Control fibroblasts (C2) were transfected with various constructs, namely, a plasmid expressing GFP-progerin, GFP-wild-type lamin A, or GFP alone, and the chromatin structure of the 14q32 locus was evaluated by 3D-FISH (Figure 2H). As anticipated, 100%, 97% and 98% of nuclei showed colocalized signals in the control conditions (nontransfected, transfected with GFP, and transfected with GFP-wild-type lamin A, respectively), whereas we observed only 72% of colocalized signals in control C2 fibroblasts transfected with the GFP-progerin plasmid (Figure 2I, p< 0.001, Fisher’s test). Moreover, the distance between the 14q32 probe and the nuclear envelope was increased under GFP-progerin conditions compared with control conditions (Figure 2J).

To assess the specificity of the chromatin remodeling observed in 14q32 locus, we investigated a subtelomeric control region, at the 1p36 locus (a region with LADs located around 5Mb from the telomeres) in C2 fibroblasts transfected with GFP-progerin, GFP-wild-type lamin A, or GFP alone plasmids. We used two probes on the 1p36 separated by approximately 1.4 Mb, as in the 14q32 experiments (Figure S2A). For this other subtelomeric region, we did not find any difference in the distance of the probes to the nuclear envelope, nor to the number of separated and colocalized probes, between the conditions GFP-progerin and GFP-wild-type lamin A (Figures S2B–S2D). These results suggest that, in our hands, the progerin modifies specifically the miRNA cluster at the 14q32 locus and seems to not have a global effect (at the LADs levels).

Finally, we assessed the local chromatin structure in C5, HGPS5, and HGPS6 fibroblasts by chromatin immunoprecipitation combined with quantification of enrichment by droplet digital PCR (ChiP-ddPCR) for different sites across the cluster, i.e.*,* miR-376a (miR-376a-1 and miR-376a-2), miR-376b, and other miRNAs (Table S2), using antibodies against CTCF and H3K36me3 shown to be enriched at this locus (UCSC genome browser [Casper et al., 2018]). We observed significant changes in CTCF and H3K36me3 enrichment in HGPS fibroblasts compared with the control (Figures S2E and S2F), reinforcing the results obtained by 3D-FISH and again, confirming the chromatin changes in this cluster.

Altogether, these observations highlight the conformational change of the 14q32 locus in HGPS fibroblasts containing genes of the overexpressed miRNAs and the role of progerin in this reorganization.

### Selection of miR-376a-3p and miR-376b-3p as candidates in HGPS pathophysiology

**Figure 3:**
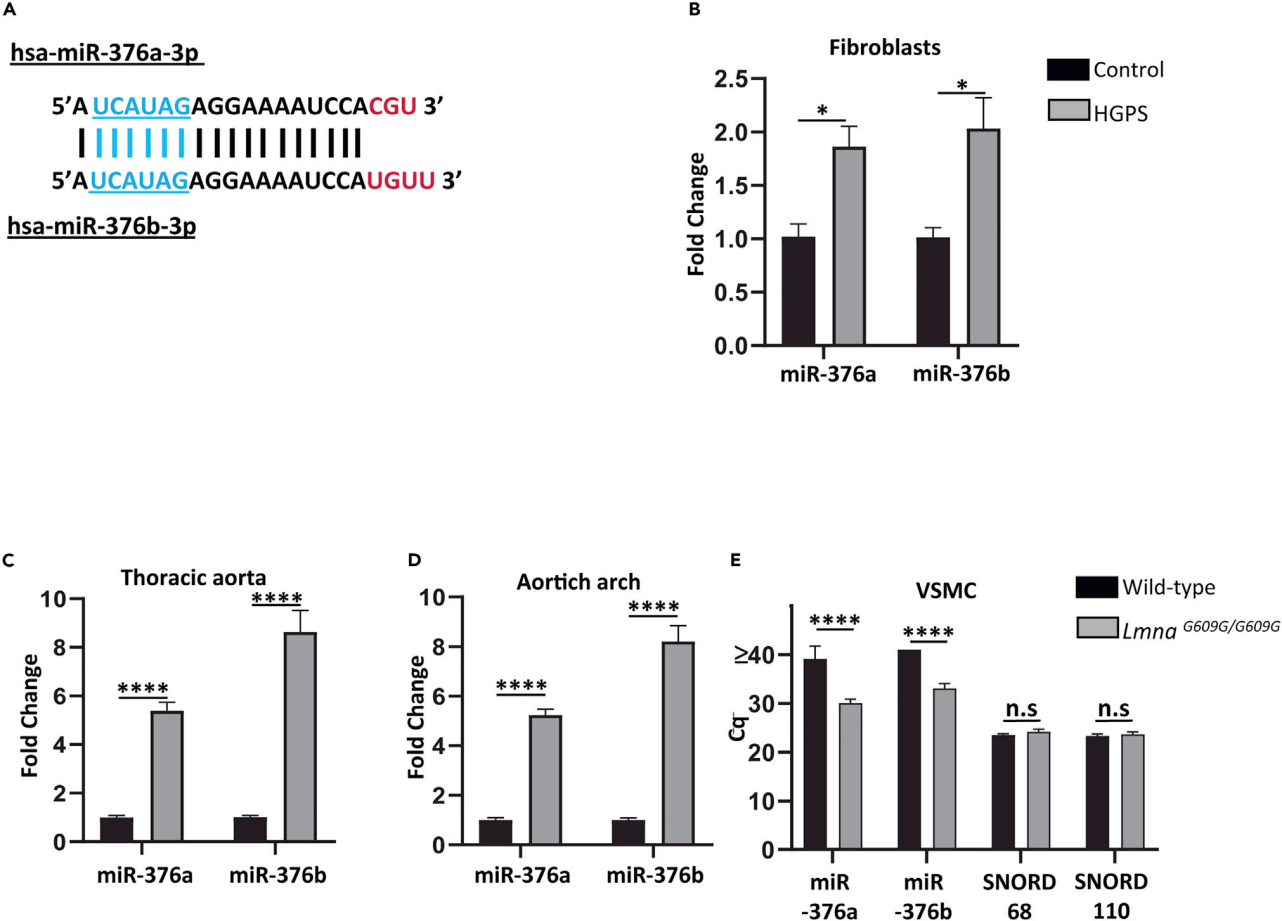
miR-376a-3p and miR-376b-3p expression in human fibroblasts and *Lmna*^*G609G/G609G*^ mouse model (A) hsa-miR-376a-3p and hsa-miR-376b-3p sequences; these two miRNAs belong to the miR-376 family and share 18 common nucleotides including the seed sequence (in blue); the last nucleotides at the 3′ end are different (in red). (B) Expression of hsa-miR-376a-3p and hsa-miR-376b-3p quantified by RT-qPCR in HGPS fibroblasts (n = 4) as compared with the control fibroblasts (n = 4, unpaired t-test). (C and D) Expression of mmu-miR-376a-3p and mmu-miR-376b-3p quantified by RT-qPCR in *Lmna*^*G609G/G609G*^ mice (n = 8) as compared with wild-type mice (n = 5) in (C) thoracic aorta and (D) aortic arch (unpaired t-test). (E) Expression (Cq) of mmu-miR-376a-3p and mmu-miR-376b-3p quantified by RT-qPCR in cultured VSMC from two aorta of *Lmna*^*G609G/G609G*^ mice (n = 6) as compared with wild-type mice (n = 4) (ANOVA test). Data are represented as mean ± SEM (∗p < 0.05, ∗∗p < 0.01, ∗∗∗p < 0.001, and ∗∗∗∗p < 0.0001).

Based on the results of miRNA expression profiling in HGPS fibroblasts, we focused our attention on the most upregulated miRNA among the 14 selected miRNAs, miR-376b-3p (FC = 2.22, Figure 1B and https://doi.org/10.6084/m9.figshare.9642803.v1.). This miRNA belongs to the miR-376 family, which includes miR-376a, miR-376b, and miR-376c, all located within the 14q32 locus. As miR-376a-3p and miR-376b-3p share a highly conserved sequence including a same seed sequence (Figure 3A), which is essential for the recognition of the target mRNAs, they might be functionally related. In the miRNA expression profiling results, miR-376a-3p was upregulated in three out of five HGPS fibroblasts (HGPS1 P13, HGPS2 P20, and HGPS5 P14), with an FC (fold change) > 2 (https://doi.org/10.6084/m9.figshare.9642803.v1.). We quantified by RT-qPCR in an independent experiment miR-376a-3p and miR-376b-3p expression in HGPS and control fibroblasts (n = 4 in each group) and confirmed that these two miRNAs were differentially expressed in HGPS fibroblasts compared with controls (Figure 3B, miR-376a-3p FC = 1.86, miR-376b-3p FC = 2.03, p< 0.05, unpaired t-test).

To evaluate the relevance of these two miRNAs in an *in vivo* context, we quantified the expression of *mmu*-miR-376a-3p and *mmu*-miR-376b-3p in the thoracic aorta and aortic arch of *Lmna*^G609G/G609G^ mice (n = 8) compared with wild-type mice (n = 5), as aorta is a tissue highly altered in this model with a loss of vascular smooth muscle cells (VSMCs). We observed overexpression of these two miRNAs in the thoracic aorta (Figure 3C, miR-376a-3p FC = 5.37, miR-376b-3p FC = 8.63, p< 0.001, unpaired t-test) and the aortic arch (Figure 3D, miR-376a-3p FC = 5.23, miR-376b-3p FC = 8.21, p< 0.001, unpaired t- test) of *Lmna*^G609G/G609G^ mice. Moreover, using freshly extracted vascular smooth muscle cells (VSMCs) from the thoracic aorta of *Lmna*^G609G/G609G^ mice (n = 6) and wild-type mice (n = 4) cultured *in vitro*, we quantified *mmu*-miR-376a-3p and *mmu*-miR-376b-3p expression using RT-qPCR. Although we did not detect either miRNA in wild-type mice, both miRNAs were expressed above detection level (Cq < 35) in *Lmna*^G609G/G609G^ mice (miR-376a-3p Cq = 30.1 ± 0.7; miR-376b-3p Cq = 33.1 ± 0.9, Figure 3E).

Altogether, these results confirm the overexpression of these two miRNAs in both *in vitro* and *in vivo* models of HGPS, suggesting their possible involvement in pathophysiology of this syndrome.

### miR-376a-3p and miR-376b-3p overexpression inhibits fibroblasts' proliferation and induces premature senescence

**Figure 4:**
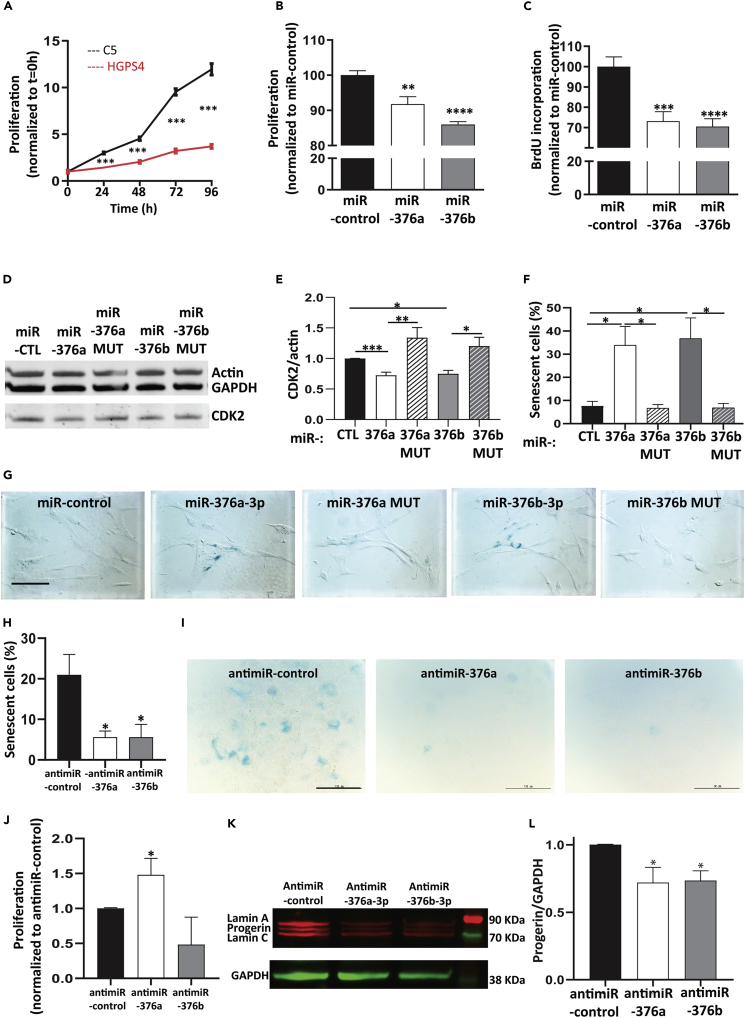
miR-376a-3p and miR-376b-3p modulate cell proliferation, senescence, and progerin expression (A) Cell proliferation of HGPS4 and C5 fibroblasts measured with CellTiter-Glo Luminescent Cell Viability Assay (ANOVA, n = 3, values are normalized to t = 0 h). (B) Cell proliferation of C5 fibroblasts measured with CellTiter-Glo Luminescent Cell Viability Assay after transfection of miR-376a-3p, miR-376b-3p, or miR-control (unpaired t-test, n = 3, values are normalized to miR-control). (C) DNA replication of C5 fibroblasts measured with BrdU relative expression after transfection of miR-376a-3p, miR-376b-3p, or miR-control (unpaired t-test, n = 4). (D and E) Western blot analysis on control fibroblast whole-cell lysates. Immunodetection of CDK2, GAPDH, and actin after miR-376a-3p, miR-376a MUT, miR-376b-3p, and miR-376b MUT transfection as compared to miR-control (n = 3 reproduced on C1 and C5, Mann Whitney test). (F and G)SA-β-gal staining of control fibroblasts transfected with miR-control, miR-376a-3p, miR-376a MUT, miR-376b-3p, and miR-376b MUT (Mann Whitney test, n = 4 for C1 and reproduced on C2 and C5). (H and I) SA-β-gal staining of HGPS fibroblasts transfected with antimiR-control, antimiR-376a-3p, or antimiR-376b-3p for 15 days (Mann Whitney test, n = 4). (J) Proliferation of HGPS fibroblasts transfected with antimiR-376a-3p, antimiR-376b-3p, or antimiR-control, for 15 days, measured with trypan blue (Mann Whitney test, n = 3). (K and L) Western blot analysis on HGPS fibroblasts whole-cell lysates, representative experiment. Immunodetection of lamin A/C, progerin, and GAPDH, after antimiR-376a-3p or antimiR-376b-3p transfection compared with antimiR-control (unpaired t-test, n = 3 on HGPS1 and HGPS5). Data are represented as mean ± SEM (∗p < 0.05, ∗∗p < 0.01, ∗∗∗p < 0.001, and ∗∗∗∗p < 0.0001).

MiR-376a-3p and miR-376b-3p are known to inhibit proliferation in different cell types (Fellenberg et al., 2016, 2019; Liu et al., 2020; Wang et al., 2011). As HGPS fibroblasts proliferate at a slower rate than controls (ANOVA, p< 0.001, Figure 4A) and undergo premature senescence (Mann Whitney test, p = 0.0286, Figure S3A), we hypothesized that miR-376a-3p and miR-376b-3p may play a role in these cellular defects observed in HGPS.

To validate our hypothesis, we first aimed to reproduce the overexpression identified in HGPS fibroblasts by transfecting these two miRNAs (independently) or a control miRNA (miR-control) into control fibroblasts, and we evaluated consequences on cell proliferation, cell cycle, and senescence.

We evaluated cell proliferation using two different tests: the CellTiter-Glo Luminescent Cell Viability Assay and the BrdU incorporation test. In both assays, transfection of miR-376a-3p or miR-376b-3p significantly decreased proliferation compared with the miR-control (Figure 4B, CellTiter-Glo Luminescent Cell Viability Assay: p = 0.0029 and p< 0.0001, respectively, unpaired t-test; Figure 4C, BrdU incorporation: p = 0.007 and p = 0.003, respectively, unpaired t-test), independently of transfection-associated toxicity (Figure S3B).

We next investigated whether miR-376a-3p and miR-376b-3p inhibit cell-cycle progression by flow cytometry. Control fibroblasts overexpressing miR-376a-3p or miR-376b-3p displayed a higher proportion of cells in G0-G1 phase and a lower proportion in G2-M phase than miR-control transfected fibroblasts, suggesting slight modulation of the cell cycle by these miRNAs (Figures S3C and S3D). As miR-376a-3p is described to directly target the CDK2 transcript, a cyclin-dependent kinase promoting G1/S transition (Wang et al., 2011), we evaluated the level of this protein by western blot. The direct effect of miR-376a-3p and miR-376b-3p on CDK2 mRNA was evaluated by transfecting in C1 and C5 fibroblasts, miR-376a-3p or miR-376b-3p mimics, or their mutant forms on seed region (i.e., abolishing the binding to CDK2 mRNA: miR-376a-3p MUT and miR-376b-3p MUT, Figure S3E). Transfection of miR-376a-3p or miR-376b-3p reduced CDK2 protein level, whereas the mutant mimics had no effect (Figures 4D and 4E), confirming the specific effect of these two miRNAs on CDK2 mRNA in our model.

We then evaluated the effect of miR-376a-3p and miR-376b-3p on senescence. The proportion of senescent cells was markedly increased after transfection of each of the two miRNAs (Figures 4F and 4G, 33.8 ±8.1% for miR-376a-3p and 36.7 ± 8.9% for miR-376b-3p versus 7.6 ± 1.9% for miR-control, n = 4, p = 0.0286, Mann-Whitney test), but not with their respective mutant forms (6.7 ± 1.6% for miR-376a-3p MUT; 6.8 ± 1.9% for miR-376b-3p MUT, n = 4, p = 0.0286, Mann-Whitney test), hence suggesting an influence of miR-376a-3p and miR-376b-3p on senescence induction.

To challenge these results, we transfected HGPS fibroblasts with antimiR-376a-3p, antimiR-376b-3p, or antimiR-control for 15 days. By inhibiting each of these miRNAs, we observed that the percentage of HGPS senescent fibroblasts was highly reduced compared with the control condition (Figures 4H and 4I, n = 4, p = 0.0159 and p = 0.0476 respectively, Mann-Whitney test). After antimiR-376a-3p transfection, these effects were associated with a significant increase of proliferation compared with antimiR-control (Figure 4J, n = 3, p = 0.0244, Mann-Whitney test), whereas the antimiR-376b-3p had no effect.

Altogether, these results indicate that the overexpression of miR-376a-3p or miR-376b-3p in fibroblasts reduces cell proliferation associated with CDK2 inhibition and induces premature senescence, which could participate in HGPS pathophysiology.

### Inhibition of miR-376a-3p or miR-376b-3p overexpression reduces progerin expression in HGPS fibroblasts

As the decrease of miR-376a-3p and miR-376b-3p in HGPS fibroblasts improves several defects of HGPS fibroblasts, we wondered if they had an impact on progerin expression. Interestingly, antimiR-376a or antimiR-376b transfection led to a decrease of progerin expression (28% ± 11 and 26.5% ± 7) (Figures 4K and 4L, p = 0.0318 and p = 0.0042 respectively, unpaired t-test), without modification of lamin A expression (data not shown).

As autophagy is involved in progerin degradation, we decided to explore if autophagy could be modulated in HGPS fibroblasts due to miR-376a-3p and miR-376b-3p overexpression.

First, we quantified autophagy under basal condition in HGPS (n = 4) and control (n = 5) fibroblasts. We assessed LC3BII protein level and presence of autophagic vacuoles (Autophagy detection Kit, Abcam). In both assays, we observed a lower level of autophagic markers in HGPS when compared with control fibroblasts, as reported by lower LC3BII levels (Figures S4A and S4B, p= 0.0052, unpaired t-test) and lower median fluorescence intensity (MFI) (Figure S4C, p = 0.047, unpaired t-test). Under induction of autophagy with rapamycin at the same concentration, LC3BII levels were increased in control fibroblasts, whereas they remain unmodified in HGPS samples (Figures S4D and S4E). In addition, treatment of HGPS fibroblasts with bafilomycin blocked autophagic flow. Altogether, these results suggest reduced basal autophagic activity in HGPS fibroblasts, with decreased activation or sensitivity to rapamycin treatment under the experimental conditions tested.

To further assess the impact of miR-376a-3p and miR-376b-3p on autophagy in HGPS fibroblasts, and evaluate autophagic flux, we used ptfLC3, an mRFP-GFP tandem fluorescent-tagged LC3 plasmid (ptfLC3) (Kimura et al., 2007). This plasmid allows discrimination of autophagosomes, which exhibit GFP and mRFP signals (corresponding to yellow punctua) before fusion with lysosomes, from autolysosomes, which only exhibit the mRFP signal. We then starved cells in HBSS medium to induce autophagy. Alternatively, we treated cells with chloroquine, an autophagic inhibitor that alters the acidic pH of lysosomes, to inhibit the fusion between autophagosomes and lysosomes, thus leading to autophagosomes accumulation. Regardless of the condition of autophagy stimulation, the number of autophagosomes increased in HGPS fibroblasts 48 h after antimiR-376a-3p or antimiR-376b-3p transfection (i.e., 24 h after ptfLC3 plasmid transfection) compared with antimiR-control transfection (Figures S4F and S4G, ANOVA test). HBSS did not significantly increase the number of autophagosomes per cell compared with the untreated condition. This result confirmed the decrease of sensitivity of HGPS fibroblasts to usual autophagy inducers, already observed after rapamycin treatment. On the other hand, and as expected, the treatment with chloroquine induced an increase of autophagosomes compared with the untreated condition. Lysosome labeling was not observed under any of the conditions tested. We confirmed these results by flow cytometry, showing an increase of the MFI after antimiR-376a-3p or antimiR-376b-3p transfection compared with antimiR-control (Figure S4H, p< 0.0001 and p = 0.0006 and, unpaired t-test) and by western blot on HGPS6 fibroblasts treated with rapamycin and bafilomycin (Figure S4I).

Altogether, these results suggest that overexpression of miR-376a-3p or miR376b-3p could participate in progerin accumulation in HGPS fibroblasts due to a decrease of its degradation by autophagy inhibition.

## Discussion

This study is the first to present the results of miRNA expression profiling in HGPS human fibroblasts. To our knowledge, only two other studies have reported miRNA expression profiling in a progeroid context, either in mouse embryonic fibroblasts derived from the murine *Zmpste24*^−/−^ model, phenotypically close to HGPS patients (Xiong et al., 2015; Zhang et al., 2017), or from *Zmpste24*^−/−^ mouse liver (Mariño et al., 2010; Ugalde et al., 2011).

We identified 14 differentially expressed miRNAs in HGPS fibroblasts compared with controls, with seven out of the eight overexpressed belonging to the same cluster at the 14q32 region. This region is an imprinted locus that contains both maternally imprinted noncoding RNAs (*MEG3*, *MEG8*, and *AS-RTL1*) and paternally imprinted genes (*DLK1*, *DIO3*, and *RTL1*). The 14q32 miRNAs are generated from a polycistronic transcript that contains the whole cluster under a coordinated regulation with MEG3 (Glazov et al., 2008; Seitz et al., 2004). Dysregulation of miRNAs belonging to this region has been described in several pathologies, mostly in cancers (Benetatos et al., 2013; Merkerova et al., 2018). Nadal et al. found a correlation between the expression of *DLK1*, *MEG3*, and the 14q32 miRNAs cluster and hypothesized a regulation by DNA methylation (Nadal et al., 2014). Among the miRNAs tested in our study belonging to the 14q32 cluster, some of them were not overexpressed. Other studies in different pathological contexts also described an heterogeneity of expression of the 14q32 cluster suggesting other regulating mechanisms (Jishnu et al., 2020; Krokker et al., 2021).

In HGPS cells, progerin accumulates with passages and induces chromatin conformational changes (Harhouri et al., 2017; Köhler et al., 2020; Lionetti et al., 2020; Robin and Magdinier, 2016; Sebestyén et al., 2020; Shumaker et al., 2006). We hypothesized that the enrichment of overexpressed miRNAs from the same region could be secondary to chromatin remodeling in HGPS fibroblasts, as supported by other studies at different loci in other contexts (McCord et al., 2013; Merkerova et al., 2018; Oldenburg et al., 2017; Wood et al., 2014). We observed that this locus is in the vicinity of the inner nuclear membrane in control fibroblasts, but distant in HGPS fibroblasts, suggesting loss of its anchoring to the nuclear *lamina*. Finally, we demonstrated that the decrease in progerin induced by AON transfection relocates the miRNA gene cluster at the nuclear envelope in HGPS fibroblasts, and consistently, overexpression of progerin in control fibroblasts localizes the miRNA gene cluster away from the nuclear envelope. Therefore, we confirmed the major role of A-type lamins in the topological organization of chromosomal territories and the deleterious effect of progerin on chromatin organization in HGPS fibroblasts nuclei. Very importantly, these results also indicate the possibility of restoring the nuclear chromosome topology by direct and targeted reduction of progerin. Indeed, the decrease of progerin should have an impact on other regions than the 14q32 region.

Among the deregulated miRNAs pointed by our study, we identified the overexpressed miR-376a-3p and miR-376b-3p as potential actors in HGPS pathophysiology, for several reasons.

First, we demonstrated that, in control fibroblasts, miR-376a-3p or miR-376b-3p ectopic overexpression decreases cell proliferation and CDK2 expression as described in hematopoietic progenitor cells (Wang et al., 2011). Consistently, in HGPS cells, the inhibition of miR-376a-3p results in an increase of cell proliferation. MiR-376a-3p is described to decrease proteins involved in cell-cycle regulation, in addition to CDK2, such as CHK1, Cyclin D2, and Cyclin A (Martinho et al., 1998; Sheng et al., 2013; Yao et al., 2018). We further demonstrated that miR-376a-3p or miR-376b-3p overexpression increases cellular senescence, therefore contributing to the accelerated aging phenotype, whereas their inhibition delays the transition into senescent state. The miR-376a-3p is already described to participate in senescence, even if its effect seems cell-type-dependent (Ye et al., 2020; Yentrapalli et al., 2015).

Moreover, we demonstrated that the inhibition of miR-376a-3p or miR-376b-3p in HGPS fibroblasts moderately but significantly decreases progerin, and that autophagy could participate in this process. Autophagy is a mechanism of degradation and recycling of cellular components, including misfolded, mutant, or abnormal proteins. Our results suggest that, under basal conditions, autophagy is decreased in HGPS fibroblasts compared with controls and is not induced after treatment with conventional simulators (starvation or rapamycin) at the incubation time and concentration tested, contrary to control fibroblasts. Our results are not inconsistent with other studies that showed an activation of autophagy using rapamycin in HGPS fibroblasts, as rapamycin was incubated for a much longer time (from 6 to 150 days) (Cao et al., 2011; Cenni et al., 2011). Therefore, we hypothesized that the incubation time is a major criteria to activate autophagy in HGPS fibroblasts, as these cells seem less sensitive to the usual autophagy activation conditions used in control fibroblasts. Using different techniques, we confirmed that miR-376a-3p or miR-376b-3p inhibition increases autophagy in HGPS fibroblasts, possibly linked to the direct targeting of mRNAs encoding major autophagic proteins, e.g., ATG4c, Beclin-1, and ATG5 (Korkmaz et al., 2012, 2013; Yang et al., 2018).

Finally, we also observed that miR-376a-3p and miR-376b-3p are overexpressed *in vivo* in aorta (aortic arch and thoracic aorta) from *Lmna*^G609G/G609G^ mice and are expressed in primary VSMCs from this tissue, whereas the VSMCs from wild-type mice do not express these miRNAs. These cells are deeply depleted in the medial layer of the aortic arch of *Lmna*^G609G/G609G^ mice (Osorio et al., 2011; Zaghini et al., 2020), a region subjected to high hemodynamic stress. These results emphasize the potential role of these miRNAs in *in vivo* vascular alteration, representing a major event conditioning the vital prognosis in this disease, which could be very interesting to explore.

Overall, our results further illustrate the complexity of HGPS pathophysiology. We propose a pathophysiological model in which the accumulation of progerin leads to epigenetic modifications associated with the overexpression of several miRNA-encoding genes located in the 14q32 cluster. Among them, miR-376a-3p and miR-376b-3p participate in cell-cycle modulation, leading to decreased proliferation and premature entry into senescence that contribute to accelerated aging at the organism level. Moreover, miR-376a-3p and miR-376b-3p overexpression amplified progerin accumulation in HGPS cells possibly by targeting transcripts encoding proteins involved in the autophagy process, inducing a vicious deleterious cycle.

### Limitations of the study

In the study presented here, we provide evidence for the decrease of progerin expression linked to miR-376a-3p or miR-376b-3p inhibition in HGPS fibroblasts. Our results suggest that autophagy is involved in this mechanism and that autophagy is different in HGPS fibroblasts compared with control. A limitation of the study is that, although we showed that miR-376a-3p or miR-376b-3p inhibition leads to autophagy activation in HGPS fibroblasts, we did not precisely dissect all the mechanisms, as autophagy process is very complex. Further experiments are required to fully understand which step is modified in HGPS fibroblasts and how miR-376a-3p and miR-376b-3p are involved. This approach would highly benefit from further improvements including mRNAs and proteins quantification of autophagic actors, including targets of miR-376a-3p and miR-376b-3p linked to this process.

## STAR★Methods

### Key resources table

**Table:** 

REAGENT or RESOURCE	SOURCE	IDENTIFIER
**Antibodies**		

anti-CDK2 Rabbit monoclonal [E304]	Abcam	Cat# ab32147; RRID:AB_726775
Anti-Actin Antibody, clone C4	Millipore	Cat# MAB1501; RRID:AB_2223041
Anti-Glyceraldehyde-3-Phosphate Dehydrogenase Antibody, clone 6C5	Millipore	Cat# MAB374; RRID:AB_2107445
MAP LC3beta (G-2) antibody	Santa Cruz Biotechnology	Cat# sc-271625; RRID:AB_10714949
Lamin A/C antibody (polyclonal)	Proteintech	Cat# 10298-1-AP; RRID:AB_2296961
SQSTM1 (D-3) antibody	Santa Cruz Biotechnology	Cat# sc-28359; RRID:AB_628279
IRDye 680RD Donkey anti-Mouse IgG antibody	LI-COR Biosciences	Cat# 926-68072; RRID:AB_10953628
IRDye 800CW Donkey anti-Rabbit IgG antibody	LI-COR Biosciences	Cat# 926-32213; RRID:AB_621848
Progerin (13A4D4) antibody	Santa Cruz Biotechnology	Cat# sc-81611; RRID:AB_1128450
Lamin A/C (H-110) antibody	Santa Cruz Biotechnology	Cat# sc-20681; RRID:AB_648154
Goat anti-Mouse IgG (H + L) Cross-Adsorbed Secondary Antibody, Alexa Fluor 488	Thermo Fisher Scientific	Cat# A-11001; RRID:AB_2534069
Goat anti-Rabbit IgG (H + L) Cross-Adsorbed Secondary Antibody, Alexa Fluor 594	Thermo Fisher Scientific	Cat# A-11012; RRID:AB_2534079
ChIPAb + Histone H3 (C-term) antibody	Millipore	Cat# 17-10046; RRID:AB_10618160
ChIPAb + Trimethyl-Histone H3 (Lys36) antibody	Millipore	Cat# 17-10032; RRID:AB_10615601
ChIPAb + CTCF Validated Antibody and Primer Set	Millipore	Cat# 17-10044; RRID:AB_10732951

**Chemicals, peptides, and recombinant proteins**		

Rapamycin	Sigma	553211
chloroquine	Abcam	ab139484
Bafilomycine A1	Sigma	5.08409
DAPI	Thermo Fisher Scientific	D1306
Ribonuclease A	Sigma	R5503
Lipofectamine™ RNAiMAX Transfection Reagent	Thermo Fisher Scientific	13778500
jetPRIME®	Polypus Transfection	114-15
Trypan blue 0.4%	Eurobio	CSTCOL03-0P

**Critical commercial assays**		

Senescence β-galactosidase Staining kit	Cell Signaling Technology	9860
BrdU Cell Proliferation ELISA Kit	Abcam	ab126556
FxCycle^TM^ Far Red Stain	Thermo Fisher Scientific	F10348
CellTox™ Green Cytotoxicity Assay	Promega	G8741
CellTiter-Glo® 2.0 Cell Viability Assay	Promega	G9241
Autophagy Assay Kit	Abcam	ab139484
Pierce™ BCA Protein Assay Kit	Thermo Fisher Scientific	23225
SYTOX™ AADvanced™ Dead Cell Stain Kit	Thermo Fisher Scientific	S10349
miRCURY LNA SYBR Green PCR Kit	Qiagen	339346
miRNeasy Mini Kit	Qiagen	217004
miRCURY LNA RT Kit	Qiagen	339340

**Deposited data**		

miRNA expression profiling	This paper	https://doi.org/10.6084/m9.figshare.9642803.v1.

**Experimental models: Cell lines**		

15-5968	CRB-TAC	N/A
13-13622	CRB-TAC	N/A
13-8243	CRB-TAC	N/A
13-13090	CRB-TAC	N/A
AG11513	Coriell	Cat# AG11513; RRID:CVCL_0Q97
GM01972	Coriell	Cat# AG01972; RRID:CVCL_F261
AG11498	Coriell	Cat# AG11498; RRID:CVCL_H766
GM08398	Coriell	Cat# GM08398; RRID:CVCL_7481
AG08498	Coriell	Cat# AG08498; RRID:CVCL_1Y51
AG07095	Coriell	Cat# AG07095; RRID:CVCL_0N66
AG08471	Coriell	Cat# AG08471; RRID:CVCL_1Y50

**Experimental models: Organisms/strains**		

C57BL/6J *Lmna*^G609G/G609G^*Mus musculus*	(Osorio et al., 2011)	N/A
C57BL/6J wild type *Mus musculus*	Charles river	N/A

**Oligonucleotides**		

miRIDIAN microRNA Human hsa-miR-376a-3p - Mimic	Horizon discovery	C-300683-03-0010
miRIDIAN microRNA Human hsa-miR-376b-3p	Horizon discovery	C-300741-03-0010
miRIDIAN microRNA Mimic Negative Control	Horizon discovery	CN-001000-01-05
miR-376a-3p_MUT: Custom miRNA Mimic, miRIDIAN mimic	Horizon discovery	CTM-549257
miR-376b-3p_MUT: Custom miRNA Mimic, miRIDIAN mimic	Horizon discovery	CTM-549261
miRCURY LNA inhibitor control, negative control B	Qiagen	YI00199007
miRCURY LNA miRNA Power Inhibitor Control, Negative control A	Qiagen	YI00199006
miRCURY LNA miRNA Power Inhibitor hsa-miR-376a-3p	Qiagen	YI04100305
miRCURY LNA miRNA Power Inhibitor hsa-miR-376b-3p	Qiagen	YI04101339
Control scrambled Ex10-scrambled 50-ATCGGCTTGTCGCGTGAGCGATCGA-3	Gene Tools	MO-HSex10scrambled
Control scrambled Ex11-scrambled 50-ACCAGTGGCGTCGCCTCGCAGGTCC-30.	Gene Tools	MO-HsEx11SCRAMBLED
exon 10-lamin A splice donor site Ex10 (50-GCTACCACTCACGTGGTGGTGATGG-30)	Gene Tools	MO-HSEX10
Ex11 (50-GGGTCCACCCACCTGGGCTCCTGAG-30) bound the c.1824C > T; p.Gly609Gly-mutated sequence in the region of the LMNA transcript	Gene Tools	MO-HSEX11
SNORD110(mmu) miRCURY LNA miRNA PCR Assay	Qiagen	YP00203912
SNORD49A (hsa) miRCURY LNA miRNA PCR Assay	Qiagen	YP00203904
hsa-miR-376b-3p miRCURY LNA miRNA PCR Assay	Qiagen	YP00204218
hsa-miR-376a-3p miRCURY LNA miRNA PCR Assay	Qiagen	YP00204508
mmu-miR-376b-3p miRCURY LNA miRNA PCR Assay	Qiagen	YP00205058
mmu-miR-376a-3p miRCURY LNA miRNA PCR Assay	Qiagen	YP00205059
Primers for ddPCR see Table S2	This paper	N/A
RainbowFish Probes RP11-123M6 (green)	Amplitech	RP11-123M6 (green)
RainbowFish Probes RP11-123M6 (orange)	amplitech	RP11-123M6 (orange)
RainbowFish Probes RP11-815P21 (red)	amplitech	RP11-815P21 (red)
**Recombinant DNA**		
ptfLC3 plasmid	(Kimura et al., 2007) Addgene	plasmid # 21074
pBABE puro EGFP plasmid	Oskar Laur, Addgene	plasmid # 128041
pBABE-puro-GFP-wt-lamin A plasmid	(Scaffidi and Misteli, 2006) Addgene	plasmid # 17662
pBABE-puro-GFP-progerin plasmid	(Scaffidi and Misteli, 2006) Addgene	plasmid # 17663

**Software and algorithms**		

R	R	https://cran.r-project.org/mirrors.html
Image Lab Software	Bio-Rad	https://www.bio-rad.com/fr-fr/product/image-lab-software?ID=KRE6P5E8Z
ZEN 2	Carl Zeiss	https://www.zeiss.com/microscopy/int/products/microscope-software/zen-lite.html
FlowJo software	FlowJo	https://www.flowjo.com/solutions/flowjo/downloads
Imaris software	Imaris	https://imaris.oxinst.com/products/imaris-for-cell-biologists
GraphPad Prism 8	GraphPad	https://www.scienceplus.com/nl/prism-commercieel.html
Exiqon GenEx® software	Exiqon	N/A

### Resource availability

#### Lead contact

Further information and requests for resources and reagents should be directed to and will be fulfilled by the lead contact, Patrice Roll (patrice.roll@univ-amu.fr).

#### Materials availability

This study did not generate new unique reagents.

### Experimental model and subject details

#### Cells

Dermal fibroblasts from HGPS patients and controls were issued from a skin biopsy, cultured and stored by the labeled Biological Resource Center (CRB TAC) (La Timone Hospital, Assistance Publique des Hôpitaux de Marseille, France) or were purchased from the Coriell Institute for Medical Research. All biological samples from CRB TAC were accompanied by signed informed consent to use them for research purposes. In the manuscript, HGPS and wild-type (control) fibroblasts are named as described in Table S1. Fibroblasts were cultured in Dulbecco’s modified Eagle’s medium low glucose (VWR, Radnor, Pennsylvanie, USA) supplemented with 15% fetal bovine serum (Thermo Fisher, Waltham, MA, USA), 2 mM L-glutamine (Thermo Fisher) and 100 U/mL penicillin, streptomycin, and amphotericin B mix (Thermo Fisher) at 37 °C in a humidified atmosphere containing 5% CO_2_.

#### Mice

Knock-in mouse model *Lmna*^*G609G/G609G*^ carrying the c.1827C > T (p.Gly609Gly) mutation (Osorio et al., 2011) and wild-type mice were used for the study. Animal experiments have been carried out in compliance with the ARRIVE (Animal Research: Reporting of *in vivo* Experiments) guidelines, the European guidelines for the care and use of laboratory animals (EU directive 2010/63/EU). All animal procedures were carried out under protocols approved by a local and national ethical committee for animal experimentation (Ministère de l’Education Nationale, de l’Enseignement Supérieur et de la Recherche; Authorization Apafis N°7404–2016102816469761 v4). Thoracic aorta and aortic arch were extracted from 4 months-old wild-type mice (n = 5, 2 males, 3 females) and *Lmna*^*G609G/G609G*^ mice (n = 8, 4 males, 4 females) for RT-qPCR. VSMC were extracted from thoracic aorta of one-month *Lmna*^*G609G/G609G*^ mice (n = 4) and wild-type mice (n = 6). To have enough VSMC to start the culture, 2 aortas were used for one culture. VSMC were cultured in DMEM-F12 (Thermofisher) supplemented with FBS (10%) and penicillin/streptomycin (1%) at 37 °C in a humidified atmosphere containing 5% CO.

### Method details

#### Transfection and treatment

Mimics (miRIDIAN microRNA Mimic: hsa-miR-376b-3p, hsa-miR-376a-3p, Mimic Negative Control #1) and mimics mutated into their seed regions (miR-376a-3p_MUT: Custom miRNA Mimic, Active: 5′-AUAAGAUAGGAAAAUCCACGU-3′ and Passenger: 5′-GUGGAUUUUCCUAUCUUAUUU-3′; miR-376b-3p_MUT: Custom miRNA Mimic, Active: 5′-AUAAGAUAGGAAAAUCCAUGUU-3′ and Passenger: 5′-CAUGGAUUUUCCUAUCUUAUUU-3′) were purchased from Horizon Discovery LTD (Cambridge, UK). miRNA inhibitors (‘antimiR’) (miRCURY LNA^TM^ miRNA Power Inhibitors hsa-miR-376a-3p and hsa-miR-376b-3p, miRCURY LNA™ microRNA inhibitor control) were purchased from Qiagen (Valencia, CA, USA). Mimics and mimicMUT (50 nM) and antimiR (25 nM) were transfected using a Lipofectamine RNAiMAX kit (Thermo Fisher) according to the manufacturer’s instructions. The percentage of transfected cells was evaluated in C5, HGPS6 and HGPS2 fibroblasts using the fluorescent antimiR control (miRCURY LNA inhibitor control, negative control B, YI00199007, Qiagen) by flow cytometry, showing 97.8 ± 0.5% fluorescent cells (data not shown). ptfLC3 was a gift from Tamotsu Yoshimori (Addgene plasmid # 21074), pBABE puro EGFP was a gift from Oskar Laur (Addgene plasmid # 128041), and pBABE-puro-GFP-wt-lamin A and pBABE-puro-GFP-progerin were a gift from Tom Misteli (Addgene plasmids # 17662 and # 17663). A total of 125 ng or 250 ng of the plasmid was transfected using a JetPRIME kit (Polypus transfection, Illkirch, France) according to the manufacturer’s instructions. Images were acquired with an Apotom 2 system (Zeiss), and at least 100 cells were examined *per* condition. Chloroquine was provided in the Autophagy Detection Kit (ab139484, Abcam), and cells were treated at 50 μM for 3 h. HBSS (Thermo Fisher) was used for starvation (3 h). Fibroblasts were treated with rapamycin (1 μM, Sigma, Lyon, France), bafilomycin (100 nM, Sigma) or DMSO for 24 h. For antisense morpholino oligonucleotide (AON) delivery, we used the endoporter system and followed manufacturer’s instructions (Gene Tools, LLC, Philomath, OR, USA) and as previously described (Harhouri et al., 2016; Osorio et al., 2011). HGPS5 fibroblasts were transfected with endoporter, scramble AON or AON targeting *LMNA* exon 10 and exon 11. Each morpholino oligonucleotide was added at a final concentration of 20μM to cell cultures. Endoporter was added at a final concentration of 6 μM. Cells were retransfected 48h later.

#### miRNA profiling by quantitative RT-PCR

We performed a miRNA profiling analysis on 5 HGPS (HGPS1 to HGPS5) and 5 control fibroblasts (C1 to C5) at passage (P) 12 ± 2. For 3 of the 5 HGPS fibroblasts (HGPS2, HGPS3, HGPS4), the analysis was also performed at P20 ± 1. Among the 5 controls, 2 were also tested at P20 ± 1 (C1 and C5). Overall, 15 samples were profiled for miRNA expression (8 HGPS and 7 control samples).

Total RNA was extracted from dermal fibroblasts using the miRNeasy kit (Qiagen) according to the manufacturer’s instructions. Samples were quantified by absorbance using a NanoDrop DN-1000 spectrophotometer (Thermo Fisher). cDNA was synthesized from 30 ng of total RNA using miRCURY LNA^TM^ Universal RT microRNA PCR, Universal cDNA Synthesis Kit II (Qiagen), according to the manufacturer’s instructions. MicroRNA expression was investigated using microRNA Ready-to-use PCR, Human panel I (Qiagen). A 384-well plate was prepared using a Biomek 3000 robot (Beckman, Brea, California, USA) and analyzed using a LightCycler 480 (Roche, Berlin, Germany). The quantification cycle (Cq) was used to calculate relative miRNA expression using interpolate calibrators present in the 384-well microRNA Human Panel I and Exiqon GenEx® software. To avoid inaccurate results, miRNAs with a Cq > 35 in at least 50% of samples were considered not expressed and were excluded from analysis. Normalization (ΔCq) was calculated using the global mean of all expressed miRNAs (Cq ≤ 35; n = 114), and miRNA relative expression (fold-change (FC)) was calculated using the 2^−ΔΔCT^ method compared to the ΔCq mean of controls (Mestdagh et al., 2009). During the last step of our miRNA selection process, we excluded miRNAs presenting a fold-change > 2 or <0.5 in at least one of the tested controls.

We performed t-tests on the normalized data (ΔCq), comparing the expression level of all miRNAs in HGPS *vs* control fibroblasts. We considered a miRNA as significantly differentially expressed if the resulting p value was <0.05. To generate the heatmaps, we also used the normalized data (ΔCq). We calculated the Euclidian distance between samples and used Ward.D2 as the agglomeration method for hierarchical clustering.

#### Quantification of individual miRNAs by quantitative RT-PCR

Expression levels of specific miRNAs were obtained using the miRCURY LNA SYBR® Green PCR Kit (Qiagen). Quantitative PCR amplifications were performed in triplicate using the primers miRCURY LNA miRNA PCR Assay for *hsa*-miR-376a-3p, *hsa*-miR-376b-3p, *mmu*-miR-376a-3p and *mmu*-miR-376b-3p (Qiagen) on a QuantStudio 5 Real-Time PCR System (Thermo Fisher). The threshold cycle (Cq) was used to calculate relative miRNA expression by the 2^−ΔΔCT^ method normalizing to *hsa*-SNORD49A expression (Qiagen), which were defined using GeNorm and NormFinder in Exiqon GenEx® software as the optimal reference for fibroblasts in our miRNome analysis. *mmu*-SNORD110 (Qiagen) was used for the normalization of mice tissues and VSMCs. For graphical representation and statistical analysis, we used a value of 41 for the Cq of WT VSMC mice although the qPCR presented no value (>40 cycles).

#### 3D DNA fish

We used a commercial probe against the most subtelomeric region of chromosome 14q (*i.e.*, 14q32: 106, 078, 392-106, 261, 579) along with a probe generated by Nick translation using the BAC clone RP11-123M6 (miRNA cluster: 100, 834, 432-100, 861, 026; CHORI) as template. Images were acquired with a confocal system (LSM 800 with Airyscan Zeiss).

Events were ranked as separated or colocalized after reconstruction of signals using IMARIS (as previously described in (Robin et al., 2014)). Images were average 3 times to improve the signal to noise ratio. Generated.lsm files with a voxel size of 0.1 μm × 0.1 μm × 0.24 μm were processed using the IMARIS software (Bitplane, AG). After 3D reconstruction we retrieved the distances between adjacent probes using their associated intensity center as set point. Colocalization was materialized by an overlap of the two reconstructed probes. Notably, this translates into a mean average of 0.5 μm for colocalization signals.

For each nucleus 3D objects (x10) were created having the same center defined as the center of the cuboid in which the object (nucleus) is inscribed. The 3D created objects correspond to the nuclear rim of the nucleus and its reduced-dimension copies (generated by IMARIS). Reduction of the object is created until the FISH probe matches the reduced envelope. The reduced dimension object was mathematically turned into equivalent sphere volume in order to obtain a distribution of the probe within the nuclear volume for each experimental condition.

In brief, for the position of probes regarding the nuclear envelope, we generated (IMARIS) series of 10 concentric zones of equal volume *per* nucleus (each zone corresponding roughly to 1 μm) and compared the mean volume ratio of nuclei of probes. After 3D reconstruction, at least 30 nuclei (60 alleles) were examined *per* condition, and the volume of the probes, distances between their gravity centers, distances to the nuclear envelope and volume of nuclei were calculated and used for statistical analysis.

Values were compared using a non-parametric Kruskal-Wallis test; populations of separated or colocalized signals were compared to the reference condition using a Chi-square test.

#### Chromatin immunoprecipitation (ChiP)

ChIP for H3, CTCF and H3K36me3 (Millipore) was performed according to the manufacturer’s instructions (TruChIP, Covaris; and Magna ChIPA/G, Millipore). ChIPs were processed using a 4 °C overnight incubation (concentration of antibodies at 1.5 μg) with 1 μL of each preparation: IP, IgG, rabbit nonimmune serum, no crosslink control, no antibody control and 1% input were used as controls for digital droplet PCR analysis (Ludlow et al., 2014). Primers were designed for each miRNA and surrounding regions enriched with histone marks using the UCSC genome browser tools (Table S2). Each PCR primer pair was tested on genomic DNA to verify specificity and efficiency. The results were normalized to inputs and to H3.

#### Proliferation and toxicity assays

Control fibroblasts were seeded at 5,000 cells *per* well in 96-well plates supplemented with 100 μL DMEM. Toxicity and proliferation rates were determined 96 h post transfection using CellTox® Green Cytotoxicity Assay and CellTiter-Glo®, respectively (Promega, Madison, WI, USA), according to the manufacturer’s instructions. Fluorescence and luminescence were measured using a GloMax® Microplate Reader (Promega).

Proliferation/replication was also evaluated 96 hours after miRNA transfection using the BrdU Cell Proliferation ELISA Kit (ab126556, Abcam, Cambridge, UK) according to the manufacturer’s instructions. In 3 wells, cells or BrdU were omitted to serve as negative controls for nonspecific binding. Absorbance was measured at 450 nm on a GloMax® Microplate Reader (Promega).

Proliferation of HGPS fibroblasts has been assessed with trypan blue and counted on kovaslide (Labelians, Nemours, France) using a microscope (Leica, Wetzlar, Germany), after 15 days of culture.

#### Cell cycle analysis

The cell cycle was analyzed in control fibroblasts using FxCycle^TM^ Far Red Stain (Thermo Fisher Scientific) 48 h post transfection. After trypsinization and centrifugation, cells were washed in PBS, centrifuged, and fixed with a fixation buffer (Biolegend, San Diego, USA) for 20 min at room temperature. Cells were washed with Intracellular Staining Permeabilization Wash Buffer (1/10, Biolegend), centrifuged and permeabilized using the same solution for 20 min at RT. After the last centrifugation, cells were resuspended in 1 mL PBS containing 1 μL FX Cycle Far Red and 5 μL Ribonuclease A (Sigma) and incubated for 30 min at RT. Cell cycle distribution was analyzed on an Attune cytometer (Thermo Fisher). Results were analyzed using FlowJo software (FlowJo software, Oregon, USA).

#### Senescence associated β-galactosidase staining

Senescence was evaluated using a Senescence β-Galactosidase Staining Kit (Cell Signaling Technology Leiden, The Netherlands) according to the manufacturer’s instructions. The percentage of stained cells was evaluated on a microscope (Leica, Wetzlar, Germany) by two independent observers using manual blind counting. At least 100 fibroblasts were randomly selected for each condition. Results are expressed graphically as the mean percentage of senescent cells.

#### Autophagy level quantification by flow cytometry

Autophagy was measured 48 h post transfection using the Autophagy Assay Kit (ab139484, Abcam) according to the manufacturer’s instructions. Sytox (1/2,000 dilution, AAdvanced^TM^ Dead Cell Stain Kit, Thermo Fisher Scientific) was added at the last step to evaluate the percentage of dead cells and measure autophagy levels specifically in living cells. Samples were assessed using an Attune cytometer (Thermo Fisher).

#### Western blot analysis

Total fibroblast proteins were extracted in a lysis buffer containing 62.5 mM Tris-HCl, 2.3% SDS, 10% glycerol, 1 mM PMSF and bromophenol blue and then sonicated three times (30 s each). Protein concentration was evaluated with a Pierce™ BCA Protein Assay Kit according to the manufacturer’s instructions (Thermo Fisher Scientific). The absorbance was measured using a UV-Visible Absorbance Module, 540–590 nm bandpass, on a GloMax® Microplate Reader (Promega).

Equal amounts of protein (20 μg) were reduced at 95 °C for 5 min by adding 5% β-mercaptoethanol and were then loaded onto 8% Bis-Tris gels (NuPAGE^TM^ precast gel, Thermo Fisher) using NuPAGE^TM^ MES SDS Running Buffer (NP0002, Thermo Fisher). After electrophoresis, proteins were electrotransferred to Immobilon-FL PVDF membranes (IPFL00010, Millipore) using Towbin Buffer with SDS 0.02%.

Membranes were blocked in Blocking Buffer for Fluorescent western blotting (Rockland Immunochemicals, Limerick, Ireland) diluted 1:1 in PBS for 1 h at room temperature and incubated overnight at 4 °C with various primary antibodies diluted in blocking buffer supplemented with 0.05% Tween 20. The primary antibodies used were anti-lamin A/C (1/2,000, 10298-1-AP, Proteintech, Manchester, UK), anti-SQSTM1 (p62, 1/500, sc-28359, Santa Cruz Biotechnology, Dallas, TX, USA), anti-LC3B (1/1,000, sc-271625, Santa Cruz Biotechnology), anti-CDK2 (1/500, ab32147, Abcam), anti-GAPDH (1/40,000, MAB374, Millipore) and anti-actin (1/20,000, MAB1501R, Millipore).

Blots were washed with PBS-T buffer (0.1% Tween 20) and incubated with IR-Dye®800CW-conjugated or IR-Dye®680RD-conjugated secondary donkey anti-rabbit or anti-mouse antibodies (LI-COR Biosciences, Lincoln, NE, USA) diluted at 1:15,000 in blocking buffer supplemented with 0.05% Tween 20 and 0.01% SDS.

All blots were imaged using a ChemiDoc MP Imaging System (Bio-Rad), and bands were quantified on Image Lab Software (Bio-Rad). GAPDH or β-actin was used as a total cellular protein loading control.

#### Immunofluorescence

Fibroblasts were seeded into 4 chamber-well slides (SPL Lifesciences, Korea). Cells were fixed for 15 minutes (RT) in a 4% paraformaldehyde +2% sucrose solution and then permeabilized for 3 minutes at RT using permeabilization buffer (0.5% Triton X-100, 50 mM NaCl, 300 mM sucrose, 20 mM HEPES pH 7.5, 3 mM MgCl2). Cells were then incubated with primary antibodies for 40 minutes at 37 °C (anti-progerin (1/50, sc-81611) and anti-lamin A/C (1/50, sc-20681), Santa Cruz Biotechnology). After washing, cells were incubated for 20 minutes at 37 °C with secondary antibodies (A11001, A11012, 1/400 Life Technologies). Nuclei were stained with DAPI for 10 minutes at room temperature (0.1 μg/mL, Thermofisher). Slides were mounted using FluorSave™ reagent (Merck Millipore) and observed on a fluorescence microscope (ApoTome.2 Zeiss).

### Quantification and statistical analysis

Statistical analysis was performed using GraphPad Prism 8 (GraphPad Software, Inc. San Diego, CA, USA). The test used and the number of replicates for each result is described in the legend of the corresponding figure. A p-value ≤ 0.05 was considered significant (∗p< 0.05, ∗∗p< 0.01, ∗∗∗p< 0.001 and ∗∗∗∗p< 0.0001).

## Data Availability

Data reported in this paper will be shared by the lead contact upon request. Data regarding the miRNome assay have been deposited at [repository “figshare”] and are publicly available as of the date of publication: https://doi.org/10.6084/m9.figshare.9642803.v1. Methods to interpret the data are detailed in the STAR Methods section of this manuscript. This paper does not report original code. Any additional information required to reanalyze the data reported in this paper is available from the lead contact upon request.

## Supporting citations

The following references appear in the supplemental information: Dou et al., 2015; Lund et al., 2014.
