# Supplementary material for: miR-376a-3p and miR-376b-3p overexpression in Hutchinson-Gilford progeria fibroblasts inhibits cell proliferation and induces premature senescence
Source: iScience. 2022 Jan 10;25(2):103757. doi: 10.1016/j.isci.2022.103757 (PMC8800101; doi:10.1016/j.isci.2022.103757)
Supplement: Document S1. Figures S1–S4 and Tables S1 and S2 [file mmc1.pdf]

iScience, Volume *25*

Supplemental information

miR-376a-3p and miR-376b-3p overexpression in Hutchinson-Gilford progeria fibroblasts inhibits cell proliferation and induces premature senescence

Diane Frankel, Valérie Delecourt, Elva-María Novoa-del-Toro, Jérôme D. Robin, Coraline Airault, Catherine Bartoli, Aurélie Carabalona, Sophie Perrin, Kilian Mazaleyrat, Annachiara De Sandre-Giovannoli, Frederique Magdinier, Anaïs Baudot, Nicolas Lévy, Elise Kaspi, and Patrice Roll

Supplemental information

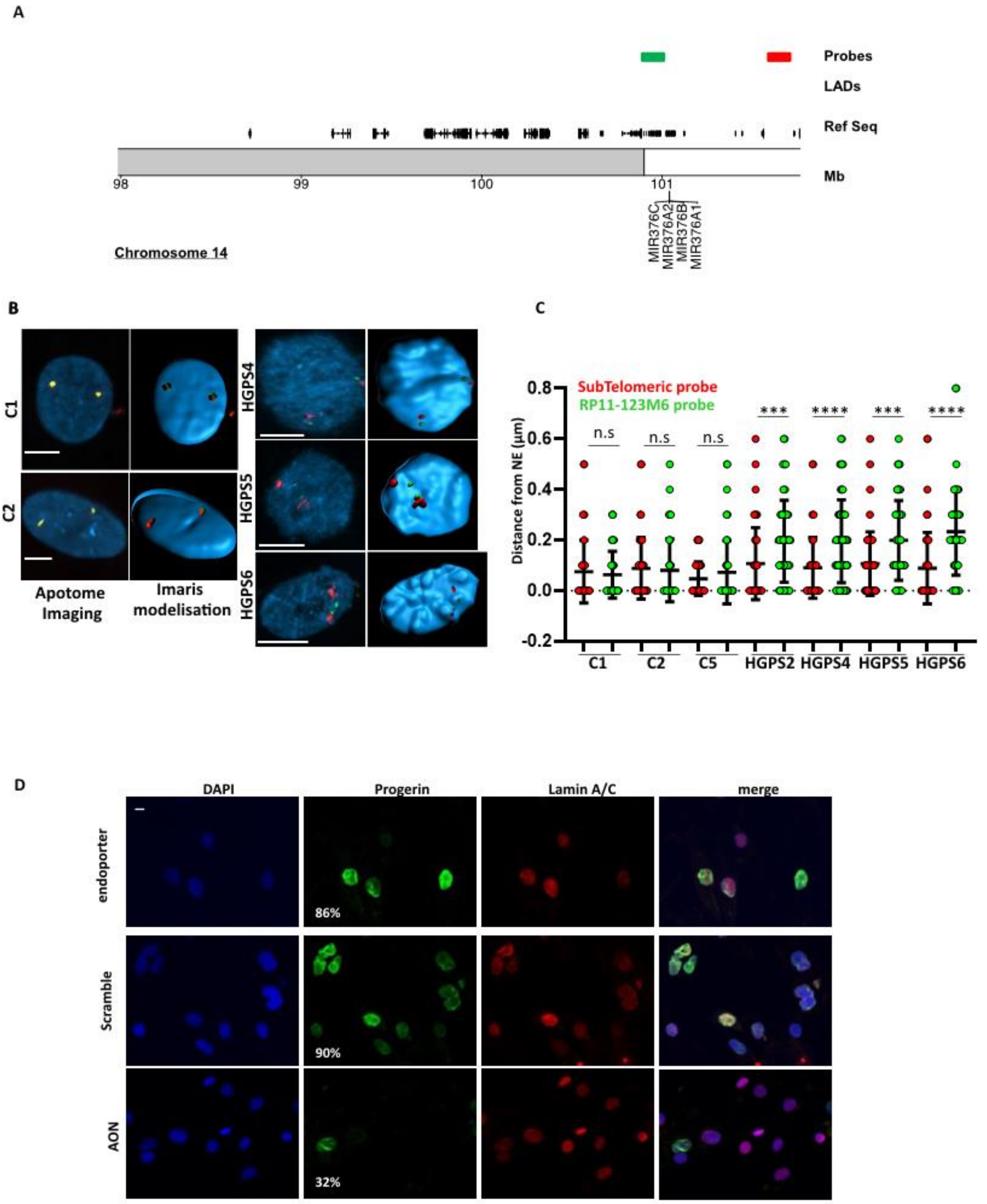

Figure S1 related to figure 2: Epigenetic modifications relative to chromosome 14 in HGPS fibroblasts.

A: Positioning of LAD and probes on chromosome 14. LADs were determined using two sets of data available : Lamin B1 (GSE63440; (Dou et al., 2015)); Lamin A (GSE54334; (Lund et al., 2014)). **B**: Representative images of 3D-FISH obtained with an apotome microscope (Carl Zeiss Microimaging, Jena, Germany) and processed with IMARIS for n≈30 nuclei *per* sample. **C**: Distance between the probes and the nuclear envelope (NE) in control fibroblasts and HGPS fibroblasts (Mann Whitney test). **D**: Immunofluorescence images of HGPS5 fibroblasts, transfected with endoporter, scramble AON (scramble) or exon 10 and exon 11 AON of *LMNA* gene (AON), stained for progerin and lamin A/C and counterstained with DAPI. Scale bar, 10 µm. Data are represented as mean +/- SEM. (*$p<0.05$, **$p<0.01$, ***$p<0.001$ and ****$p<0.0001$).

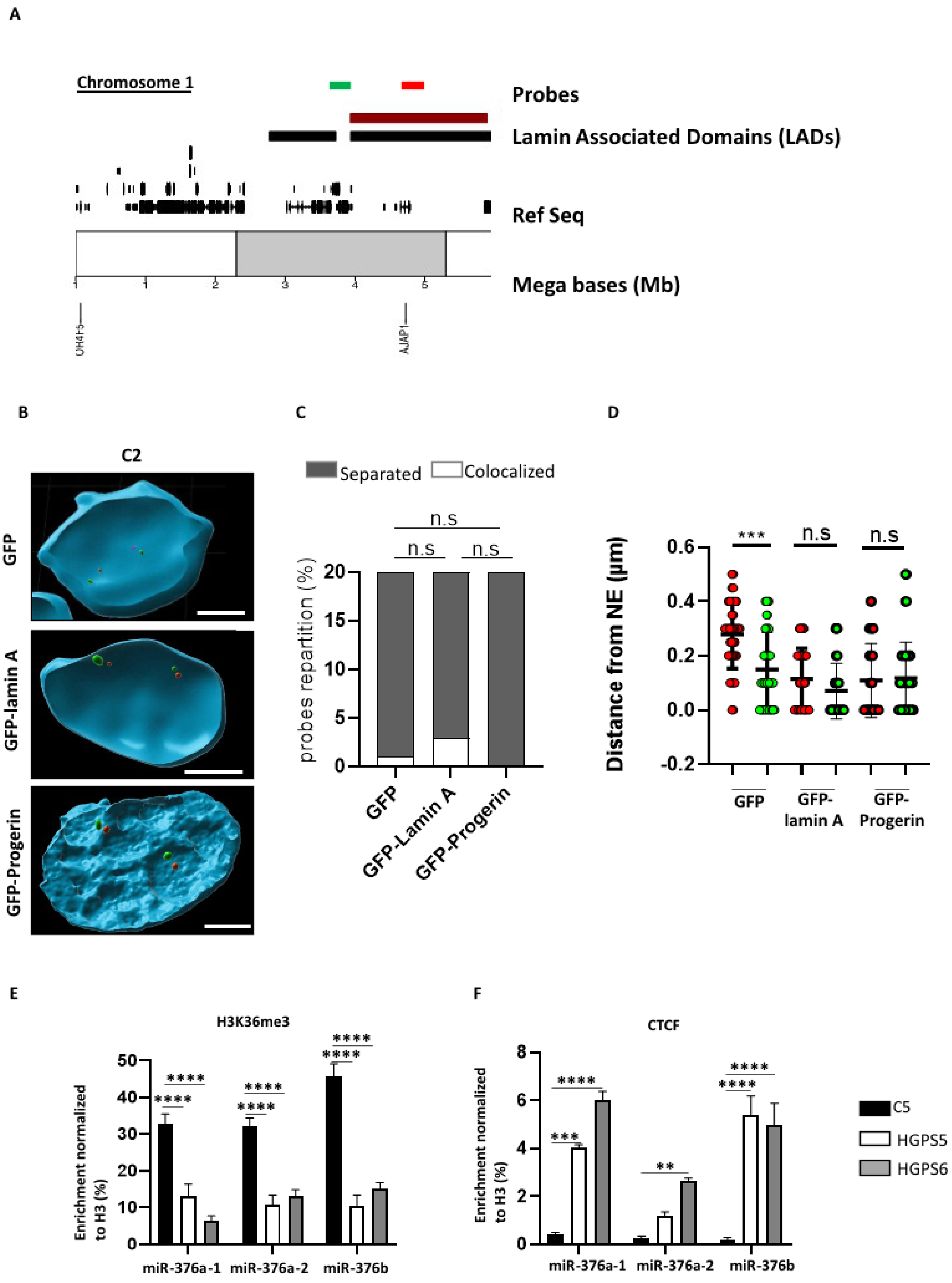
A
Chromosome 1
Probes
Lamin Associated Domains (LADs)
Ref Seq
Mega bases (Mb)
1
2
3
4
5
AJAP1
B
C2
GFP
GFP-lamin A
GFP-Progerin
C
Separated
Colocalized
n.s
n.s
n.s
probes repartition (%)
0
5
10
15
20
GFP
GFP-Lamin A
GFP-Progerin
D
Distance from NE (µm)
***
n.s
n.s
0.6
0.4
0.2
0.0
-0.2
GFP
GFP-lamin A
GFP-Progerin
E
H3K36me3
Enrichment normalized to H3 (%)
0
10
20
30
40
50
****
miR-376a-1
miR-376a-2
miR-376b
F
CTCF
Enrichment normalized to H3 (%)
0
2
4
6
8
***
**
****
miR-376a-1
miR-376a-2
miR-376b
C5
HGPS5
HGPS6

Figure S2 related to figure 2: Epigenetic modifications.

A: Positioning of LAD and probes on chromosome 1. LADs were determined using two sets of data available: Lamin B1 (GSE63440; (Dou et al., 2015)); Lamin A (GSE54334; (Lund et al., 2014). Probes were designed, a subtelomeric LADs and separated from the same genetic distance as the 14q32 probes (≈ 1.4Mb). **B**: Representative images of 3D-FISH of C2 fibroblasts transfected with a plasmid expressing only GFP (GFP), or GFP-wild-type lamin A (lamin A) GFP-progerin (progerin). Images were obtained with an apotome microscope (Carl Zeiss Microimaging, Jena, Germany) and processed with IMARIS for n≈10 nuclei per sample. Scale bar, 5µm. **C**: Percentage of separated and colocalized probes (Chi-square test). **D**: Distance between the 1p36 probes and the nuclear envelope (NE) (Mann-Whitney test). **E** and **F**: Enrichment of (**E**) H3K36me3 and (**F**) CTCF in C5, HGPS5 and HGPS6 fibroblasts on 3 different loci that belong to the 14q32 cluster (ANOVA test). Data are represented as mean +/- SEM. (*p<0.05, **p<0.01, ***p<0.001 and ****p<0.0001).

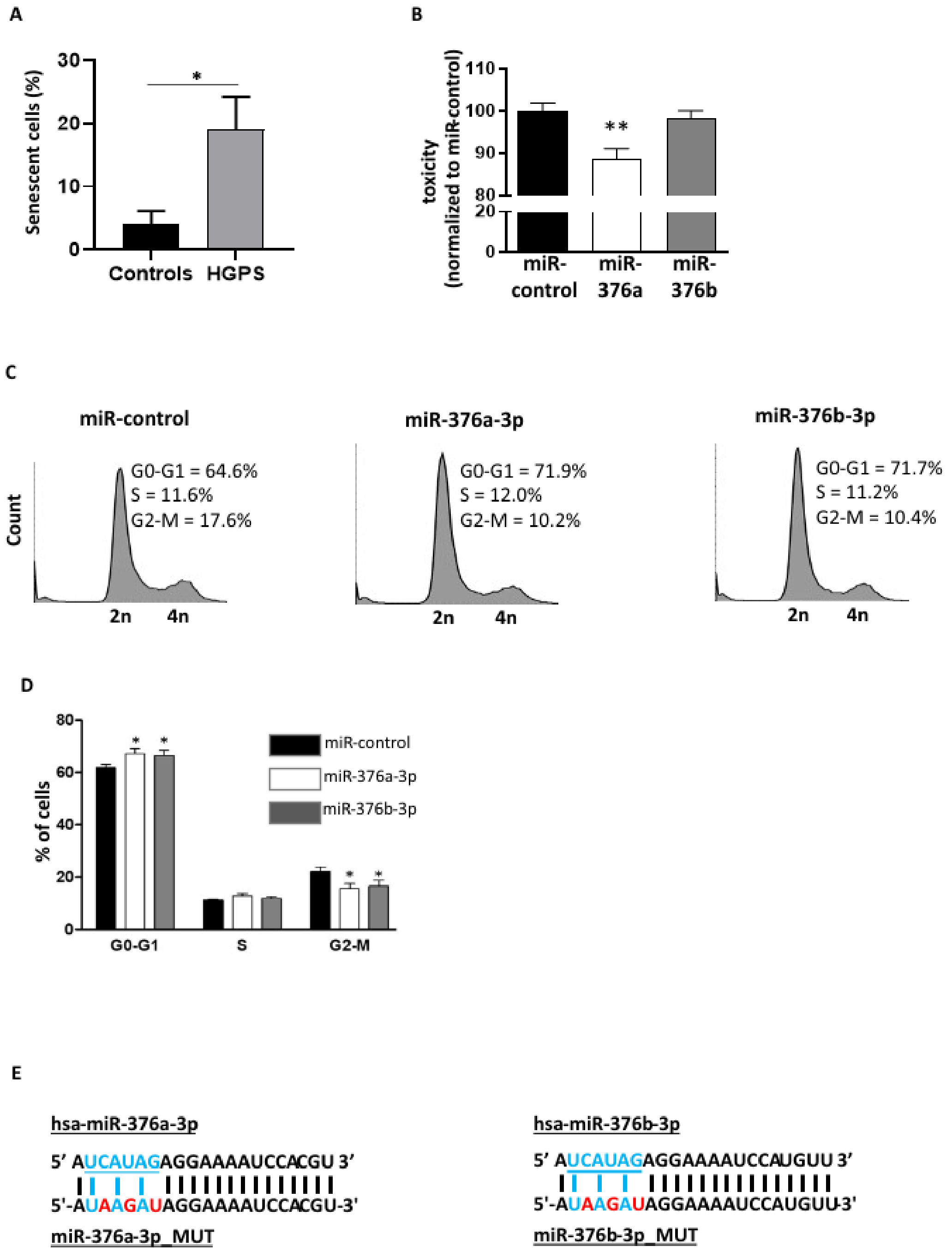

A
Senescent cells (%)
30
20
10
0
*
Controls
HGPS
B
toxicity
(normalized to miR-control)
110
100
90
80
20
0
**
miR-control
miR-376a
miR-376b
C
miR-control
G0-G1 = 64.6%
S = 11.6%
G2-M = 17.6%
miR-376a-3p
G0-G1 = 71.9%
S = 12.0%
G2-M = 10.2%
miR-376b-3p
G0-G1 = 71.7%
S = 11.2%
G2-M = 10.4%
Count
2n
4n
D
% of cells
80
60
40
20
0
miR-control
miR-376a-3p
miR-376b-3p
G0-G1
S
G2-M
E
hsa-miR-376a-3p
5’ AUCAUAGAGGAAAAUCCACGU 3’
5'-AUAAGAUAGGAAAAUCCACGU-3'
miR-376a-3p_MUT
hsa-miR-376b-3p
5’ AUCAUAGAGGAAAAUCCAUGUU 3’
5'-AUAAGAUAGGAAAAUCCAUGUU-3'
miR-376b-3p_MUT

Figure S3 related to figure 4: Basal senescent state in control and HGPS fibroblasts; effect of miR-376a-3p or miR-376b-3p overexpression on cell cycle in control fibroblasts.

A: Percentage of senescent cells in control (n=4) and HGPS (n=3) fibroblasts (Mann Whitney test). **B:** Cell toxicity measured with Cell Tox® kit (Unpaired t-test, n=3). Results are normalized with control condition (miR-control). **C** and **D**: Cell cycle analysis in C5 fibroblasts. (**C**): Representative flow cytometry pattern; (**D**): The histogram displays the percentage changes of G0-G1, S and G2-M when cells are transfected with miR-376a-3p, miR-376b-3p or miR-control (Mann-Whitney test, one-tailed, n=3). **E**: hsa-miR-376a-3p and miR-376a-3p MUT, hsa-miR-376b-3p and miR-376b-3p MUT sequences; the seed sequence is in blue and the differences between the miRNA and the mutant form are in red. Data are represented as mean +/- SEM. (*p<0.05, **p<0.01, ***p<0.001 and ****p<0.0001).

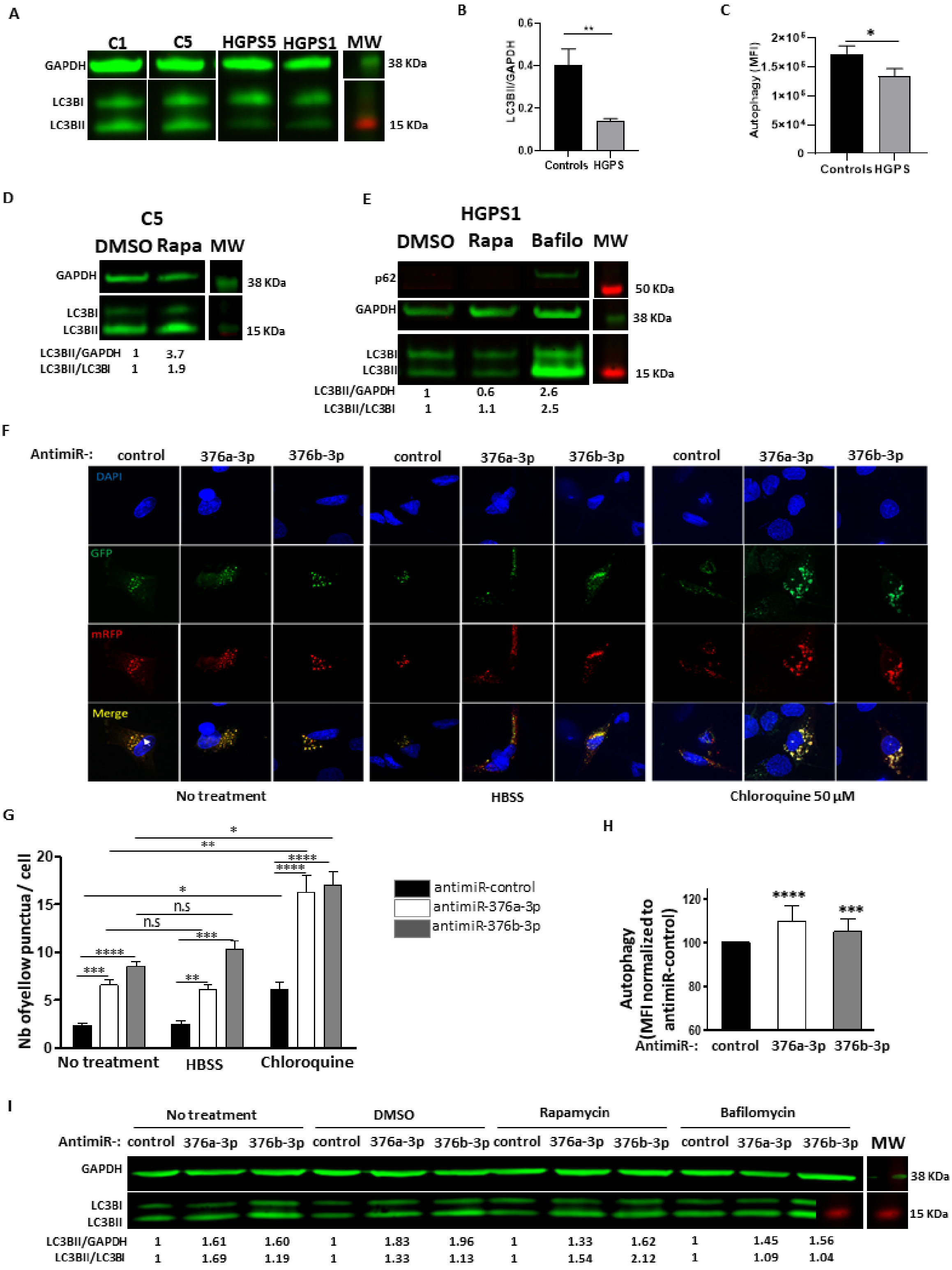
A
C1 C5 HGPS5 HGPS1 MW
GAPDH
LC3BI
LC3BII
38 KDa
15 KDa
B
LC3BII/GAPDH
Controls HGPS
C
Autophagy (MFI)
Controls HGPS
D
C5
DMSO Rapa MW
GAPDH
LC3BI
LC3BII
38 KDa
15 KDa
LC3BII/GAPDH 1 3.7
LC3BII/LC3BI 1 1.9
E
HGPS1
DMSO Rapa Bafilo MW
p62
GAPDH
LC3BI
LC3BII
50 KDa
38 KDa
15 KDa
LC3BII/GAPDH 1 0.6 2.6
LC3BII/LC3BI 1 1.1 2.5
F
AntimiR-: control 376a-3p 376b-3p control 376a-3p 376b-3p control 376a-3p 376b-3p
DAPI
GFP
mRFP
Merge
No treatment
HBSS
Chloroquine 50 µM
G
Nb of yellow punctua / cell
No treatment
HBSS
Chloroquine
antimiR-control
antimiR-376a-3p
antimiR-376b-3p
H
Autophagy (MFI normalized to antimiR-control)
AntimiR-: control 376a-3p 376b-3p
I
No treatment
DMSO
Rapamycin
Bafilomycin
AntimiR-: control 376a-3p 376b-3p control 376a-3p 376b-3p control 376a-3p 376b-3p control 376a-3p 376b-3p
MW
GAPDH
LC3BI
LC3BII
38 KDa
15 KDa
LC3BII/GAPDH 1 1.61 1.60 1 1.83 1.96 1 1.33 1.62 1 1.45 1.56
LC3BII/LC3BI 1 1.69 1.19 1 1.33 1.13 1 1.54 2.12 1 1.09 1.04

Figure S4 related to figure 4: Monitoring of basal autophagy in control and HGPS fibroblasts and role of miR-376a-3p or miR-376b-3p in autophagy.

A and **B**: Western blot analysis on fibroblast whole cell lysates. Immunodetection and quantification of LC3BI, LC3BII and GAPDH (on 5 controls and 4 HGPS fibroblasts, unpaired t-test). **C:** Basal autophagy level, represented by the mean fluorescence intensity (MFI), performed by flow cytometry (on 3 controls and 3 HGPS fibroblasts, unpaired t-test) (MW: molecular weight). **D**: Representative immunodetection of LC3BII, LC3BI, p62 and GAPDH in HGPS1 fibroblasts treated with DMSO, rapamycin (Rapa, 1µM) or bafilomycin (Bafilo, 100nM) for 24h**.** This experiment has been reproduced on HGPS2, HGPS5, HGPS6 (MW: molecular weight). **E:** Representative immunodetection of LC3BII, LC3BI and GAPDH in C5 fibroblasts treated with DMSO or rapamycin (Rapa, 1µM) for 24h (MW: molecular weight). **F and G**: HGPS6 fibroblasts transiently transfected with antimiRNAs and ptfLC3 plasmid and analyzed with fluorescence microscope (obj x63); (**F**) Representative images. **(G)** The histogram shows autophagosomes quantification (n = 3, ANOVA test) **H:** MFI normalized to antimiR-control after transfection of antimiR-376a-3p or antimiR-376b-3p (n=5, on 3 HGPS fibroblasts, unpaired t-test). **I**: Western blot analysis on fibroblast whole cell lysates. Immunodetection of LC3BI, LC3BII and GAPDH in HGPS6 fibroblasts, not treated, treated with DMSO or rapamycin (1µM) or bafilomycin (100nM). Each condition has been transfected with antimiR-control or antimiR-376a-3p or antimiR-376b-3p (MW: molecular weight). Data are represented as mean +/- SEM. (*p<0.05, **p<0.01, ***p<0.001 and ****p<0.0001).

	Patients						Controls (wild-type)				
**Source**	Coriell	CRB TAC	CRB TAC	Coriell	Coriell	CRB TAC	CRB TAC	Coriell	Coriell	Coriell	Coriell
**ID**	AG 11513	13-5968	13-13622	AG 01972	AG 11498	13-8243	13-13090	AG 07095	AG 08471	GM 08398	AG 08498
**Corresponding name**	HGPS1	HGPS2	HGPS3	HGPS4	HGPS5	HGPS6	C1	C2	C3	C4	C5
**Passage tested for miRNome study**	P13	P11 P20	P11 P20	P14 P21	P11	/	P11 P19	P11	P14	P10	P12 P20
**Age (year)**	8	2 months	8 months	14	14	2	7	2	NA	8	1
**Sexe**	female	female	male	female	male	female	male	male	male	male	male
**Fibroblasts source**	skin, leg	skin, arm	skin, arm	skin, thorax	skin, thigh	skin, arm	skin, arm	skin, foreskin	skin, thorax abdomen	skin, inguinal area	skin, foreskin

Table S1 related to STAR methods: Identification and source of fibroblasts. All HGPS patients carry the p.Gly608Gly mutation in *LMNA* (heterozygous NM_170707.4:c.1824C>T). NA: not available.

miR	Forward (F)/Reverse (R)	Sequence
**miR-300**	F	CTCTCACCATGCAGATCCCA
	R	GGAGTCAGCCTTGTTCCCAA
**miR-376a1**	F	TCGTCTGCCTCATGTGACTT
	R	TGTGTCTGTCCGTCCTGTAC
**miR-376a2**	F	GCTGCTTTGAAAACCTCGGA
	R	ATTATGTGTGCACCAAGGGC
**miR-376b**	F	TTTGTCCTTTCCAGAGCCCA
	R	GCCCTACGGTCTCTTCCAG
**miR-376c**	F	TGCTTAGGTTCATGCTTTCCAG
	R	ACCGACTTTCCACTTACCCT
**miR-654**	F	CAAATGCTGCCTTGGGATCA
	R	GACAACACACCACAGCCTG
**miR-1185-1**	F	CAGAGAGAGTTGGCCCATGA
	R	CTCCTGGTGAACACTGGCTA
**miR-1185-2**	F	AGATGAGGCTTGTCACCGG
	R	ACGCAAATGAGAGTCTCCCC

Table S2 related to STAR methods: List of primers used for ChiP-ddPCR.
